# Virus-encoded circular RNAs in human cancer: mechanisms and clinical implications

**DOI:** 10.3389/fcimb.2026.1909628

**Published:** 2026-10-01

**Authors:** JunPing Hu, Meini Li, Jingyue Zhang

**Affiliations:** Department of Clinical Laboratory, Guigang Clinical Medical Research Center for Medical Laboratory, Guigang City People’s Hospital, Guigang, Guangxi, China

**Keywords:** Epstein-Barr virus, human papillomavirus, Kaposi sarcoma-associated herpesvirus, liquid biopsy, oncogenesis, virus-encoded circRNAs

## Abstract

Circular RNAs (circRNAs) are covalently closed RNA molecules generated mainly through back-splicing. Their resistance to exonuclease-mediated degradation, cell-type specificity, and regulatory versatility make them important participants in both host and viral biology. Since the initial demonstration that circular DNA tumor viruses produce abundant circRNAs, an expanding repertoire of virus-encoded circular RNAs (viro-circRNAs) has been identified in Epstein-Barr virus (EBV), Kaposi sarcoma-associated herpesvirus (KSHV), human papillomavirus (HPV), hepatitis B virus (HBV), hepatitis C virus (HCV), and related animal tumor viruses. Accumulating evidence indicates that viro-circRNAs are not passive transcriptional by-products; rather, they function as miRNA sponges, RNA-binding protein scaffolds, modulators of RNA stability and transcription, epigenetic or epitranscriptomic regulators, and in selected cases, templates for viral peptide or oncoprotein translation. Recent studies have refined this field by revealing noncanonical herpesvirus-driven circRNA biogenesis, EBV circRPMS1-mediated METTL3 activation and p53 interaction, hypoxia-induced EBV circLMP2A-driven angiogenesis, HPV16 circE7-mediated immune evasion through promoter histone modification, HBV-derived circRNA signaling in hepatocellular carcinoma, and HCV-derived circRNAs that support viral RNA amplification. In this review, we synthesize current evidence using a mechanistic and clinically oriented framework: first defining viro-circRNA biogenesis and validation, then summarizing virus- and cancer-specific expression patterns, followed by a detailed analysis of direct tumor-cell mechanisms, tumor microenvironment remodeling, biomarker potential, therapeutic opportunities, and unresolved challenges. This framework is intended to move the discussion beyond descriptive cataloguing and toward testable hypotheses for translational viro-circRNA research.

## Introduction

Circular RNAs constitute a conserved and abundant class of RNA molecules characterized by a covalently closed loop structure generated when a downstream splice donor is joined to an upstream splice acceptor (Kristensen et al., 2019; Patop et al., 2019). This circular configuration confers resistance to exonucleolytic degradation and distinguishes circRNAs from linear mRNAs and long noncoding RNAs. Once regarded as rare splicing errors, circRNAs are now recognized as widespread regulatory molecules with tissue-specific expression, developmental regulation, and disease relevance (Salzman et al., 2012; Memczak et al., 2013; Rybak-Wolf et al., 2015; Piwecka et al., 2017).

The functional repertoire of circRNAs is broad. Many circRNAs act as competitive endogenous RNAs by sequestering miRNAs and thereby relieving repression of downstream mRNAs (Hansen et al., 2013). Others function as decoys or scaffolds for RNA-binding proteins, influence transcription, modulate mRNA stability, or participate in subcellular localization of regulatory complexes (Abdelmohsen et al., 2017). In addition, selected circRNAs can be translated through internal ribosome entry site-dependent or N6-methyladenosine (m6A)-driven cap-independent mechanisms (Legnini et al., 2017; Pamudurti et al., 2017; Yang et al., 2017). These features make circRNAs well suited to participate in cancer biology, where stable and context-specific regulatory RNAs can shape proliferation, apoptosis, invasion, immune escape, and therapy resistance (Zebardast et al., 2023).

A major conceptual shift occurred in 2018, when Toptan et al. showed that circular DNA tumor viruses, including EBV and KSHV, generate circular RNAs (Toptan et al., 2018). Subsequent studies expanded the EBV circRNA repertoire to transcripts arising from the BART/RPMS1, LMP2, BHLF1, and EBNA-related regions (Toptan et al., 2018; Ungerleider et al., 2018). KSHV encodes multiple circRNAs, including kcirc3, kcirc29, kcirc38, kcirc54, circvIRF4, and circPANs (Tagawa et al., 2018; Abere et al., 2020). HPV16 produces circE7, a protein-coding circRNA that can give rise to the E7 oncoprotein (Zhao et al., 2019). Comparative analysis of gammaherpesviruses revealed both conserved and species-specific viral circRNAs (Ungerleider et al., 2019), while more recent work demonstrated noncanonical circRNA biogenesis driven by alpha- and gamma-herpesviruses (Dremel et al., 2025). Reviews published in 2024–2025 further highlighted how pathogen-encoded circRNAs participate in viral replication, immune regulation, and therapeutic vulnerability (Kamali et al., 2024; Mueller et al., 2025).

The current challenge is no longer simply to list viral circRNAs, but to classify their biological functions, evaluate their cancer specificity, and determine which candidates are mature enough for clinical translation. Following the logic of pathway-centered reviews, this article summarizes viro-circRNA biogenesis and validation, then reviews expression and functional evidence across major oncogenic viruses, analyzes mechanisms of action, and finally discusses biomarkers, therapeutics, knowledge gaps, and future priorities.

## Biogenesis and validation of virus-encoded circRNAs

Viro-circRNAs are generated by back-splicing within viral transcripts, but their biogenesis cannot be assumed to mirror host circRNA production. EBV circRNAs arise from both latency-associated and lytic genomic regions, including BART/RPMS1, LMP2, BHLF1, and EBNA-related transcripts (Toptan et al., 2018; Ungerleider et al., 2018). KSHV circRNAs are differentially regulated across latent and lytic phases, with circvIRF4 enriched during latency and circPANs associated with lytic replication (Tagawa et al., 2018; Abere et al., 2020; Pagtalunan et al., 2025; Torres et al., 2025). Dremel et al. recently showed that herpesviruses can drive noncanonical circRNA biogenesis through mechanisms distinct from host circRNA production, emphasizing virus-specific cis-elements, altered splice-site usage, and infection-state-dependent regulation (Dremel et al., 2025). This finding is important because it suggests that viral circularization may represent a regulated component of the viral transcriptome rather than stochastic transcriptional noise.

Rigorous validation is essential. Candidate viro-circRNAs should be confirmed by back-splice-junction-specific RT-qPCR or targeted RNA sequencing, resistance to RNase R digestion, divergent primer amplification, Sanger sequencing of the junction, and, where feasible, northern blotting or long-read sequencing to define full-length isoforms. For functional studies, loss-of-function tools should target the back-splice junction without disrupting cognate linear viral transcripts. This distinction is particularly important for viral loci such as HPV16 E7 or EBV LMP2, where circular and linear transcripts may share exonic sequences but have different biological functions.

Subcellular localization also informs function. Nuclear viro-circRNAs such as EBV circEBNA_U may participate in viral promoter usage and transcript architecture, whereas cytoplasmic viro-circRNAs such as circRPMS1, circLMP2A, HPV16 circE7, HBV-circ-1, and HBV-circRNA-5 are more likely to regulate miRNA networks, RNA-binding proteins, translation, and signaling pathways (Toptan et al., 2018; Ungerleider et al., 2018; Liu et al., 2019; Qiao et al., 2019; Zhao et al., 2019; Gong et al., 2020; Zhu et al., 2021; Du et al., 2022; Zhang et al., 2022; Yang et al., 2025). Extracellular or vesicle-associated viro-circRNAs, including virion-packaged KSHV circRNAs and exosomal EBV circLMP2A, are particularly relevant to liquid biopsy development (Abere et al., 2020; Gong et al., 2026).

## Expression patterns and cancer associations

A pathway-centered review should integrate expression, prognosis, and mechanism. The evidence for viro-circRNAs in cancer is strongest for EBV-associated epithelial malignancies, KSHV-associated tumors and lymphomas, HPV-associated epithelial cancers, and HBV- or HCV-related liver disease. Across these settings, the level of evidence ranges from discovery sequencing and cell-line validation to functional assays, xenograft models, patient-tissue associations, and early liquid-biopsy feasibility (**Table 1**).

**Table 1:** Representative virus-encoded circRNAs in human cancer and related viral disease models.

Virus	circRNA	Disease/model association	Major evidence and function	Clinical or translational implication	Refs.
EBV	ebv-circRPMS1	NPC, EBVaGC, EBV-positive tumors	Multi-miRNA sponge; promotes proliferation and metastasis; interacts with Sam68 to activate METTL3; interacts with p53 in EBVaGC.	Tissue-based prognostic candidate; potential target for junction-specific knockdown.	(Liu et al., 2019; Qiao et al., 2019; Zhang et al., 2022; Yang et al., 2025)
EBV	ebv-circLMP2A/B	EBVaGC, Burkitt lymphoma, EBV-positive gastric cancer specimens	Promotes stemness through miR-3908/TRIM59/p53; hypoxia-induced KHSRP/VHL/HIF1alpha/VEGFA loop promotes angiogenesis.	Candidate marker of metastasis, stemness, angiogenesis, and exosomal liquid biopsy feasibility.	(Toptan et al., 2018; Huang et al., 2019; Gong et al., 2020; Du et al., 2022; Gong et al., 2026)
EBV	circBART family	PTLD, NPC, EBV-positive cell lines	Latency- and context-specific circRNA production from BART region.	Useful for mapping EBV latency programs and tumor-specific viral transcriptomes.	(Toptan et al., 2018; Ungerleider et al., 2018; Huang et al., 2019)
EBV	circBHLF1	Lytic EBV replication, EBV-positive lymphoma models	Derived from lytic origin-associated BHLF1 region; contains potential ORF.	Model for lytic-phase circRNA function and possible circRNA-derived viral peptides.	(Toptan et al., 2018)
EBV	circEBNA_U	EBV-positive epithelial and lymphoid contexts	Spans EBNA1-U junction; associated with Cp promoter-initiated transcript diversity.	Candidate nuclear circRNA for viral promoter and episomal transcription studies.	(Toptan et al., 2018; Ungerleider et al., 2018)
EBV	circBART2.2	NPC	Binds RIG-I and regulates PD-L1 expression; promotes immune escape.	Potential immune-checkpoint-related biomarker and therapeutic target.	(Ge et al., 2021)
KSHV	kcirc3, kcirc29, kcirc38, kcirc54	PEL, Kaposi sarcoma-associated contexts	Identified in infected tumor tissues and PEL lines; some viral circRNAs are incorporated into virions.	Potential markers of infection state and early regulatory molecules in newly infected cells.	(Tagawa et al., 2018; Abere et al., 2020)
KSHV	circvIRF4	PEL; latent and lytic KSHV infection	Latency-associated circRNA; modulates host and viral gene expression across infection phases.	Candidate regulator of KSHV persistence and replication-state biology.	(Abere et al., 2020; Pagtalunan et al., 2025; Torres et al., 2025)
KSHV	circPANs	KSHV lytic replication	Lytic-phase circRNA species derived from PAN-associated region.	Potential marker of lytic activation.	(Tagawa et al., 2018; Pagtalunan et al., 2025; Torres et al., 2025)
KSHV	circK7.3	KSHV virions and infection models	Packaged into viral particles.	Possible delivery of pre-formed regulatory RNA to newly infected cells.	(Abere et al., 2020)
MHV68/rLCV	Conserved and unique gammaherpesvirus circRNAs	Animal models of gammaherpesvirus infection	Comparative analysis reveals conserved and species-specific circRNA repertoires.	Useful models for evolutionary conservation and experimental validation.	(Ungerleider et al., 2019; Dremel et al., 2025)
HPV16	circE7	Cervical cancer; HPV-positive HNSCC	m6A-modified and translation-competent; produces E7 oncoprotein; can regulate immune escape through ACC1-H3K27ac-LGALS9 signaling.	High-interest biomarker/target candidate, but abundance and assay specificity remain debated.	(Zhao et al., 2019; Yu and Zheng, 2022; Ge et al., 2024)
HBV	HBV_circ_1	HBV-related HCC	Interacts with CDK1; promotes proliferation, migration, invasion, and tumor growth.	Candidate oncogenic HBV-derived circRNA in HCC.	(Zhu et al., 2021)
HBV	HBV-circRNA-5	HBV-related HCC	Acts through miR-9-3p/Hippo signaling; increases YAP/TAZ output and promotes tumor progression.	Potential diagnostic and therapeutic candidate requiring clinical validation.	(Yang et al., 2025)
HCV	HCV-derived circRNAs	HCV-infected cells; chronic infection model	Translated and nontranslated virus-derived circRNAs support viral RNA abundance and amplification.	Extends viro-circRNA biology to RNA viruses and chronic infection-associated disease.	(Cao et al., 2024)

ACC1, acetyl-CoA carboxylase 1; EBV, Epstein-Barr virus; EBVaGC, EBV-associated gastric carcinoma; HCC, hepatocellular carcinoma; HCV, hepatitis C virus; HBV, hepatitis B virus; HNSCC, head and neck squamous cell carcinoma; HPV, human papillomavirus; KSHV, Kaposi sarcoma-associated herpesvirus; NPC, nasopharyngeal carcinoma; PEL, primary effusion lymphoma; PTLD, post-transplant lymphoproliferative disease.

### EBV-encoded circRNAs

EBV is a ubiquitous oncogenic gammaherpesvirus etiologically linked to Burkitt lymphoma, Hodgkin lymphoma, post-transplant lymphoproliferative disease, nasopharyngeal carcinoma (NPC), and EBV-associated gastric carcinoma (EBVaGC) (O'Neil et al., 2008; Murphy et al., 2009; Dawson et al., 2012; Camargo et al., 2014; The Cancer Genome Atlas Research Network, 2014; Young et al., 2016; Lin et al., 2018; Huang et al., 2019). EBV encodes latent membrane proteins, Epstein-Barr nuclear antigens, EBERs, viral miRNAs, and a diverse circRNA repertoire (O'Neil et al., 2008; Dawson et al., 2012; Young et al., 2016; Lin et al., 2018; Huang et al., 2019). Among EBV circRNAs, circRPMS1 and circLMP2A currently have the strongest functional evidence in cancer.

EBV circRPMS1 was initially characterized as a multi-miRNA sponge that can regulate host miRNAs involved in cell-cycle progression, oncogenesis, proliferation, and metastasis (Liu et al., 2019; Qiao et al., 2019). In NPC cells, circRPMS1 knockdown suppresses proliferation and metastatic phenotypes (Liu et al., 2019). In EBVaGC, circRPMS1 promotes tumor progression through Sam68-dependent activation of METTL3, connecting viral circRNAs to m6A-mediated epitranscriptomic remodeling (Zhang et al., 2022). More recent evidence indicates that ebv-circRPMS1 can interact with p53 and that circRPMS1-p53 co-expression correlates with clinical progression and poor survival in EBVaGC (Yang et al., 2025). These data support a model in which circRPMS1 integrates ceRNA activity, protein interaction, and tumor-suppressor pathway modulation.

EBV circLMP2A provides a complementary example. It is detected in EBV-positive Burkitt lymphoma and gastric cancer specimens (Toptan et al., 2018; Huang et al., 2019). Functionally, circLMP2A promotes stemness in EBVaGC through the miR-3908/TRIM59/p53 axis (Gong et al., 2020). Under hypoxic conditions, HIF1alpha induces ebv-circLMP2A expression; circLMP2A then binds KHSRP and facilitates VHL mRNA degradation, stabilizing HIF1alpha and promoting VEGFA-mediated angiogenesis (Du et al., 2022). This positive-feedback loop links the hypoxic tumor microenvironment to viral circRNA-mediated neovascularization, metastasis, and poor prognosis. In 2026, exosomal profiling of EBVaGC cancer stem cell-derived exosomes further showed enrichment of ebv-circLMP2A and association with metastatic phenotypes, supporting its potential as an extracellular-vesicle biomarker candidate (Gong et al., 2026).

The immune context of EBVaGC is also important when interpreting these viral circRNA mechanisms. EBV-positive gastric tumors commonly display an immune-rich phenotype with prominent CD8+T-cell infiltration together with increased immune-checkpoint activity, including PD-L1 overexpression (The Cancer Genome Atlas Research Network, 2014). This coexistence of active cytotoxic-cell recruitment and checkpoint restraint provides a clinically relevant setting in which EBV circRNAs may influence tumor–immune crosstalk. CircRPMS1- and circLMP2A-dependent signaling can plausibly reinforce tumor persistence by integrating epitranscriptomic remodeling, p53 modulation, stemness, and hypoxia-driven angiogenesis (Gong et al., 2020; Du et al., 2022; Zhang et al., 2022; Yang et al., 2025). However, direct evidence that individual EBV circRNAs control CD8+T-cell states in EBVaGC remains limited; future studies should therefore test correlations among viro-circRNA abundance, PD-L1 expression, cytotoxic T-cell functional states, and response to immune-checkpoint blockade.

Additional EBV circRNAs broaden the functional landscape. circBART families show latency- and tumor-context-specific expression, circBHLF1 is associated with lytic replication and contains a potential open reading frame, and circEBNA_U is linked to Cp promoter-initiated transcript diversity (Toptan et al., 2018; Ungerleider et al., 2018). EBV circBART2.2 promotes immune escape in NPC by regulating PD-L1, providing an immune-checkpoint-related mechanism with potential predictive relevance (Ge et al., 2021).

### KSHV-encoded circRNAs

KSHV/HHV-8 is the etiological agent of Kaposi sarcoma, primary effusion lymphoma, and multicentric Castleman disease. KSHV circRNAs were identified in primary effusion lymphoma cell lines and infected tumor tissues, and some are incorporated into virions (Tagawa et al., 2018; Abere et al., 2020). Virion packaging implies that viral circRNAs may be delivered to newly infected cells before *de novo* viral transcription begins, providing a mechanism for early regulatory effects.

KSHV circvIRF4 is latency-associated and contributes to viral persistence (Abere et al., 2020). circPANs are abundant during lytic replication (Tagawa et al., 2018). Recent studies summarized by Pagtalunan et al. and mechanistic work by Torres et al. indicate that KSHV circRNAs can modulate both viral and host gene expression during latent and lytic replication (Pagtalunan et al., 2025; Torres et al., 2025). At present, the clinical biomarker evidence for KSHV circRNAs is less mature than for EBV circRNAs, but their infection-phase specificity and virion incorporation make them attractive candidates for studying KSHV replication status, tumor biology, and immune evasion (Abere et al., 2020; Media et al., 2025; Pagtalunan et al., 2025; Torres et al., 2025).

### HPV-encoded circRNAs

High-risk HPV types, especially HPV16 and HPV18, drive cervical cancer and contribute to anogenital and oropharyngeal cancers (Zheng and Baker, 2006; Lowy and Schiller, 2012). HPV16 circE7 is the best-characterized HPV-encoded circRNA. It is m6A-modified, cytoplasmic, associated with polysomes, and capable of producing E7 oncoprotein, thereby contributing to pRb degradation and cell-cycle deregulation (Zhao et al., 2019). This discovery expanded the concept of viro-circRNAs from noncoding regulators to stable templates for viral oncoprotein translation.

The current research status of circE7 should be discussed cautiously. One study questioned the abundance and functional dominance of HPV16 circE7 in cervical cancer cells, suggesting that circE7 may be low relative to linear HPV transcripts and that assay specificity is critical (Yu and Zheng, 2022). More recent work in HPV-positive head and neck squamous cell carcinoma showed that circE7 promotes immune evasion by binding ACC1, reducing H3K27 acetylation at the LGALS9 promoter, suppressing galectin-9 expression, and altering immune-checkpoint-associated T-cell interactions (Ge et al., 2024). Thus, HPV16 circE7 remains a high-interest candidate, but its clinical utility requires standardized back-splice-junction-specific assays and validation in adequately powered patient cohorts (Sindhu et al., 2026).

### HBV- and HCV-derived circRNAs

In liver disease, it is important to distinguish host circRNAs induced by viral infection from bona fide virus-encoded circRNAs. HBV_circ_1 was identified as an HBV-derived circular RNA that interacts with CDK1 and promotes hepatocellular carcinoma proliferation, migration, invasion, and tumor growth (Zhu et al., 2021). HBV-circRNA-5, reported in 2025, promotes HCC progression through a miR-9-3p/Hippo signaling axis, leading to increased YAP/TAZ pathway output (Yang et al., 2025). These examples suggest that HBV-derived circRNAs may directly contribute to hepatocarcinogenesis rather than merely reflecting chronic infection.

HCV provides an important RNA-virus example. HCV-infected cells generate virus-derived circRNAs, including translated and nontranslated species that can enhance viral RNA abundance and support viral amplification (Cao et al., 2024). The HCV findings broaden viro-circRNA biology beyond DNA tumor viruses and indicate that viral genome type, replication compartment, and RNA-processing environment shape circRNA production and function.

### DNA/RNA-level mechanisms of viro-circRNA action

As in pathway-based oncologic reviews, viro-circRNA mechanisms can be divided into direct tumor-cell effects and indirect effects mediated through the tumor microenvironment. The available evidence indicates that viro-circRNAs converge on a limited set of cancer hallmarks: sustained proliferation, resistance to apoptosis, p53 pathway attenuation, angiogenesis, stemness, immune evasion, and metastatic dissemination.

### miRNA sponging and ceRNA rewiring

The ceRNA model remains one of the most established mechanisms. EBV circRPMS1 contains multiple miRNA response elements and can sequester host miRNAs, thereby derepressing mRNAs involved in oncogenic signaling (Liu et al., 2019; Qiao et al., 2019). This activity is linked to NPC proliferation and metastasis (Liu et al., 2019). EBV circLMP2A acts through the miR-3908/TRIM59/p53 axis to support EBVaGC stemness (Gong et al., 2020). HBV-circRNA-5 binds miR-9-3p and activates Hippo pathway output through YAP/TAZ regulation (Yang et al., 2025). Importantly, viro-circRNA-mediated miRNA sequestration should not be treated as a generic explanation; it must be demonstrated in each cellular context by stoichiometry, direct binding assays, rescue experiments, and downstream target validation.

### RNA-binding protein scaffolding and RNA stability control

Protein interaction mechanisms are increasingly important because they can operate even when circRNA abundance is modest. EBV circRPMS1 interacts with Sam68 to activate METTL3, thereby linking viral circRNAs to m6A-dependent epitranscriptomic remodeling (Zhang et al., 2022). It also interacts with p53, providing a direct route to tumor-suppressor pathway modulation (Yang et al., 2025). EBV circLMP2A binds KHSRP and enhances VHL mRNA degradation, resulting in HIF1alpha stabilization and VEGFA induction (Du et al., 2022). HBV_circ_1 binds CDK1 and promotes malignant behavior in HCC (Zhu et al., 2021). These examples demonstrate that viro-circRNAs can act as scaffolds, decoys, or adaptors that redirect host proteins toward viral or oncogenic regulatory programs.

### Transcriptional, epigenetic, and epitranscriptomic regulation

The epigenetic role of viro-circRNAs should be stated precisely. Direct evidence that viro-circRNAs recruit DNA methyltransferases or Polycomb complexes to silence classical tumor suppressor genes remains limited. However, several studies support a model in which viro-circRNAs regulate cancer-relevant chromatin or RNA-modification states. HPV16 circE7 provides the strongest direct chromatin-level example: by binding ACC1 and reducing H3K27 acetylation at the LGALS9 promoter, circE7 suppresses galectin-9 expression and contributes to immune escape in HPV-positive head and neck cancer (Ge et al., 2024). EBV circRPMS1 activates METTL3 through Sam68, linking viral circRNA expression to m6A-dependent post-transcriptional regulation (Zhang et al., 2022). Nuclear EBV circEBNA_U is associated with Cp promoter-initiated transcript diversity, suggesting possible cis-regulatory effects on viral transcription, although direct chromatin-isolation and RNA-protein interaction studies are still needed (Li et al., 2015; Toptan et al., 2018; Ungerleider et al., 2018).

### Translation of viral peptides and oncoproteins

Translation-competent viro-circRNAs represent a distinctive mechanism. HPV16 circE7 can be translated into E7 oncoprotein, which retains transforming activity through pRb degradation (Zhao et al., 2019). EBV circBHLF1 contains a potential open reading frame, and HCV-derived circRNAs containing viral internal ribosome entry site elements may be translated into viral products with proviral functions (Toptan et al., 2018; Cao et al., 2024). These findings show that a viro-circRNA can act simultaneously as a stable RNA molecule and as a protein-coding template. Future work should define the prevalence, abundance, antigenicity, and functional contribution of circRNA-derived viral peptides in patient tumors.

### Tumor microenvironment remodeling and immune escape

Viro-circRNAs can also reshape the tumor microenvironment. EBV circLMP2A links hypoxia to angiogenesis through the KHSRP/VHL/HIF1alpha/VEGFA axis (Du et al., 2022). EBV circBART2.2 promotes PD-L1-mediated immune escape in NPC (Ge et al., 2021). HPV16 circE7 suppresses galectin-9 through H3K27ac reduction at LGALS9 and alters T-cell immune interactions (Ge et al., 2024). KSHV circRNAs are involved in latent and lytic transcriptional programs and may contribute to innate immune evasion in gammaherpesvirus-associated disease (Media et al., 2025; Pagtalunan et al., 2025; Torres et al., 2025). These findings suggest that viro-circRNAs may influence not only tumor cells but also endothelial recruitment, cancer stem cell maintenance, immune-checkpoint signaling, and antiviral immune surveillance. In EBVaGC, the characteristic combination of high CD8+ T-cell infiltration and PD-L1 overexpression provides an especially relevant immunological context in which such circRNA-dependent mechanisms could help convert an immune-rich microenvironment into a functionally restrained one. Together, these processes extend viro-circRNA biology beyond tumor-intrinsic signaling to endothelial recruitment, cancer stem-cell maintenance, immune-checkpoint control, and antiviral immune surveillance.

## Clinical implications

### Diagnostic and prognostic biomarkers

The circular structure and virus-specific back-splice junctions of viro-circRNAs provide theoretical advantages for biomarker development. Compared with host circRNAs, viro-circRNAs have greater tumor specificity in virus-associated cancers because their sequences are absent from uninfected human cells. EBV circRPMS1 is upregulated in EBV-positive NPC and gastric carcinoma tissues and is associated with metastatic behavior and poor clinical outcome (Liu et al., 2019; Zhang et al., 2022; Yang et al., 2025). EBV circLMP2A is linked to stemness, angiogenesis, metastasis, and exosomal enrichment in EBVaGC models (Gong et al., 2020; Du et al., 2022; Gong et al., 2026). circBART2.2 may serve as an immune-evasion marker in NPC through PD-L1 regulation (Ge et al., 2021). HPV16 circE7 has tissue and transcriptomic support but requires careful interpretation because of concerns about abundance and assay specificity (Zhao et al., 2019; Yu and Zheng, 2022; Ge et al., 2024). HBV-circ-1 and HBV-circRNA-5 are promising candidates in HBV-related HCC, but their clinical application requires separation from host-derived circulating circRNAs (Zhu et al., 2021; Yang et al., 2025).

Despite this promise, the clinical evidence remains immature. Most studies have used tumor tissues, cell lines, xenograft sera, or small patient cohorts. Large prospective plasma, serum, and extracellular-vesicle studies are still needed. Future biomarker studies should include standardized sample handling, RNase R controls, actinomycin D stability assays, absolute quantification of back-splice junctions, matched viral DNA and linear viral transcript measurements, and independent training-validation cohorts.

### Therapeutic targeting

The most attractive therapeutic feature of viro-circRNAs is the presence of virus-specific back-splice junctions. Antisense oligonucleotides, siRNAs, locked nucleic acid gapmers, or CRISPR-Cas13 systems could theoretically suppress circular viral transcripts while sparing host linear transcripts and uninfected cells. Proof-of-principle support comes from circRPMS1 knockdown, which suppresses NPC proliferation and metastasis (Liu et al., 2019), and from IL-like pathway logic in oncology, where neutralizing a disease-promoting molecular axis can restore treatment sensitivity or remodel the tumor microenvironment. For viro-circRNAs, however, delivery, off-target effects, impact on linear viral transcripts, and tumor heterogeneity remain major challenges.

Combination strategies may be particularly relevant. In EBVaGC and NPC, viro-circRNA targeting could be combined with immune-checkpoint blockade if circRNAs regulate PD-L1, galectin-9, or T-cell suppression (Ge et al., 2021; Ge et al., 2024). In HBV- and HCV-related liver disease, viral circRNA targeting may need to be integrated with antiviral therapy and HCC surveillance. In HPV-positive tumors, strategies that target circE7 must account for the dominant contribution of linear E6/E7 transcripts and require careful assay-guided patient selection (Zhao et al., 2019; Yu and Zheng, 2022; Ge et al., 2024).

## Knowledge gaps and future directions

Several gaps define the next stage of viro-circRNA research. First, many viral circRNAs are annotated by sequencing but lack functional validation; priority should be given to candidates with strong expression, cancer specificity, conserved back-splice junctions, and patient-tissue relevance. Second, mechanistic studies must distinguish circular-transcript effects from those of cognate linear viral RNAs through junction-specific perturbation, rescue experiments, and orthogonal validation. Third, the field needs standardized clinical validation. Blood-based viro-circRNA assays should be tested prospectively in healthy controls, virus carriers without cancer, premalignant disease, and confirmed virus-associated tumors, with serial sampling before and after surgery, radiotherapy, chemotherapy, immunotherapy, or antiviral treatment to determine whether viro-circRNAs track tumor burden, residual disease, recurrence, or treatment resistance.

A particularly important unresolved question is the crosstalk between viro-circRNAs and endogenous host circRNA/miRNA/mRNA networks. Viral and host circRNAs may compete for overlapping pools of miRNAs and RNA-binding proteins, allowing viro-circRNAs to “hijack” or rebalance global ceRNA circuitry rather than acting only through isolated one-circRNA/one-miRNA axes (Torres et al., 2021; Kamali et al., 2024). Such competition could amplify oncogenic signaling by redistributing regulatory miRNAs or RBPs away from host tumor-suppressive networks, while infection-induced changes in host circRNA expression may further reinforce the same circuitry. Future studies should therefore quantify viral and host circRNAs within the same network models and combine matched RNA-seq/small-RNA-seq with perturbation-rescue experiments, single-cell analysis, or spatial profiling to determine whether this network rewiring is causal and cell-type specific.

Direct epigenetic evidence also remains limited. HPV16 circE7 has been linked to H3K27ac reduction at LGALS9, and EBV circRPMS1 activates METTL3 (Zhang et al., 2022; Ge et al., 2024), but direct demonstration that viro-circRNAs induce DNA methylation-dependent or Polycomb-dependent silencing of classical tumor-suppressor genes in patients is lacking. Chromatin isolation by RNA purification, CUT&RUN, ChIRP-seq, CLIP-seq, and single-cell multi-omics could clarify whether nuclear viro-circRNAs directly shape episomal or host chromatin. Therapeutic development is likewise still early. The most rational intervention candidates should meet five criteria: confirmed circularity, high tumor specificity, defined molecular partners, functional necessity *in vitro* and *in vivo*, and evidence of clinical association. A mechanism–localization–clinical-readiness scoring model may help distinguish mature biomarker or therapeutic candidates from early descriptive discoveries.

## Conclusion

Virus-encoded circular RNAs have expanded the conceptual landscape of viral gene regulation and virus-associated oncogenesis. Current evidence indicates that viro-circRNAs can function through miRNA sequestration, protein scaffolding, RNA stability control, transcriptional modulation, epigenetic or epitranscriptomic remodeling, immune regulation, and viral peptide or oncoprotein translation. EBV circRPMS1 and circLMP2A, HPV16 circE7, HBV_circ_1, HBV-circRNA-5, KSHV circRNAs, and HCV-derived circRNAs collectively illustrate how distinct viruses exploit circular RNA biology to regulate latency, replication, oncogenic signaling, immune escape, stemness, angiogenesis, and metastasis (**Figure 1**).

**Figure 1:**
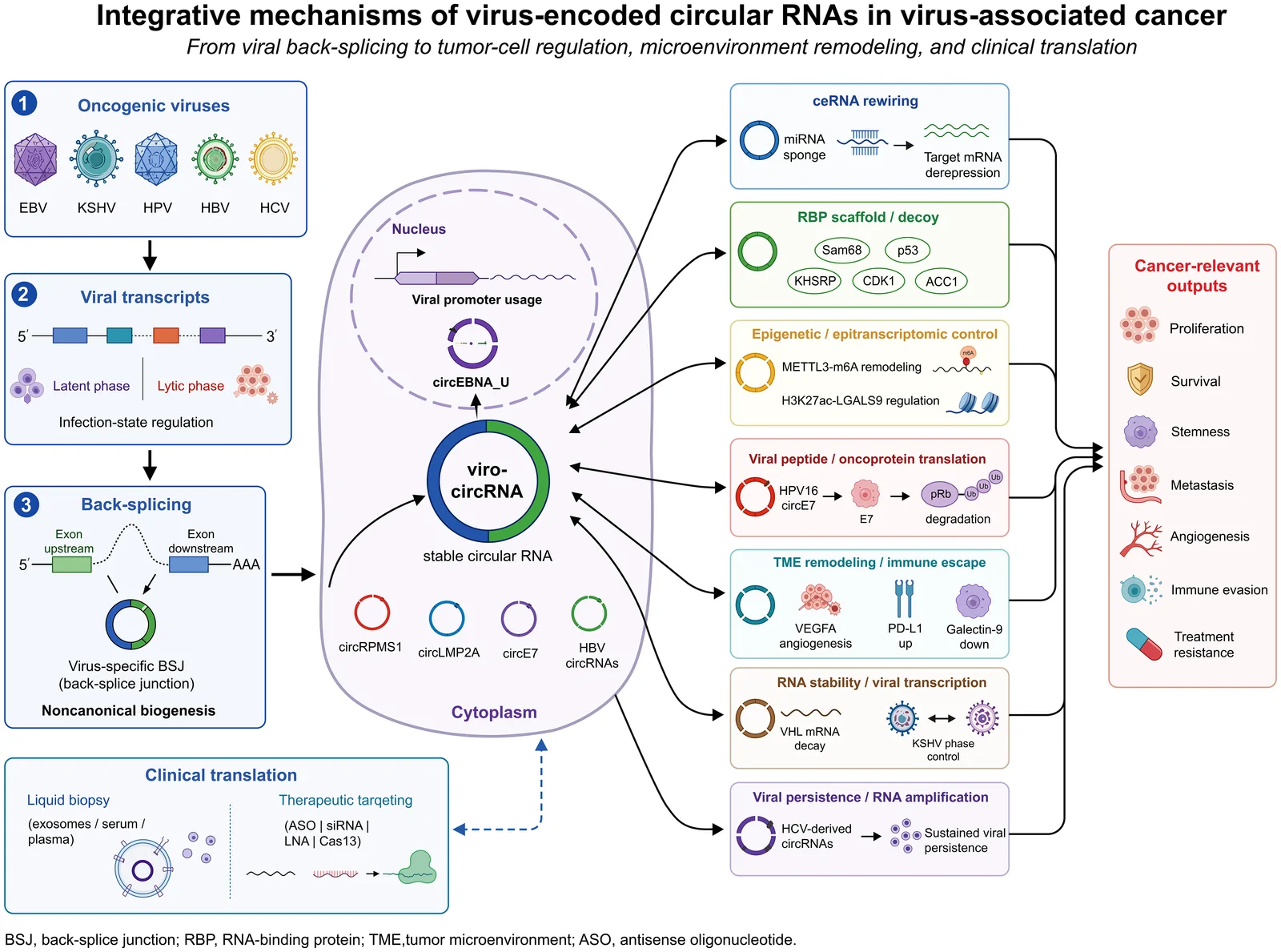
Integrative mechanisms of virus-encoded circular RNAs in virus-associated cancer. Virus-encoded circular RNAs (viro-circRNAs) are generated from viral transcripts through back-splicing and infection-state-dependent regulation. After formation, viro-circRNAs regulate virus-associated cancer through multiple mechanisms, including ceRNA-mediated miRNA sequestration, RNA-binding protein scaffolding or decoy activity, RNA stability control, epigenetic and epitranscriptomic remodeling, viral peptide or oncoprotein translation, tumor microenvironment remodeling, immune escape, and viral persistence. These mechanisms converge on cancer-relevant phenotypes such as proliferation, survival, stemness, metastasis, angiogenesis, immune evasion, and treatment resistance. Extracellular or exosomal viro-circRNAs may further serve as liquid-biopsy biomarkers, while back-splice junction-specific targeting provides potential therapeutic opportunities.

To facilitate translational prioritization, viro-circRNAs can be conceptually categorized based on their subcellular localization, core biological functions, molecular mechanisms, and clinical readiness. This classification, while not intended as a rigid framework, provides a practical heuristic for distinguishing early-stage discoveries from candidates with higher translational potential.

The field is now moving from discovery to translational prioritization. The most important future tasks are to standardize viro-circRNA detection, validate clinical associations in blood and tissue cohorts, define direct molecular mechanisms, and develop junction-specific therapeutic strategies. By organizing viro-circRNAs according to mechanism, localization, disease association, and clinical readiness, future studies can move beyond descriptive catalogues and establish testable frameworks for precision diagnosis and treatment of virus-associated cancers.
